# Targeting RhoC by Way of Ribozyme Trangene in Human Breast Cancer Cells and its Impact on Cancer Invasion

**DOI:** 10.4021/wjon2010.01.1202

**Published:** 2010-02-01

**Authors:** Jane Lane, Tracey A Martin, Wen G Jiang

**Affiliations:** a Metastasis and Angiogenesis Research Group, Cardiff University School of Medicine, Cardiff, UK

**Keywords:** RhoC, Invasion, Ribozyme, Molecular targeting, Breast cancer

## Abstract

**Background:**

Cell motility and migration are known to be regulated by the Rho family of GTPases through their effects on the actin cytoskeleton. In breast cancer studies, RhoC has been identified as a highly specific marker in detecting tumors that developed metastases. This study aims to investigate the impact of targeting RhoC in human breast cancer cells by utilising ribozyme transgene technology and to assess its effect on cancer cell invasion.

**Methods:**

Retroviral hammerhead ribozyme transgenes, regulated by doxycycline, were designed to specifically target human RhoC mRNA. The breast cancer cell line MDA-MB-231 was transfected with either a retroviral RhoC transgene or a control retroviral transgene. Stably transfected cells were tested for their invasiveness and migratory properties *in vitro*.

**Results:**

*In vitro* testing of the invasiveness of wild type, plasmid control and the RhoC knockdown cells showed that MDA-MB-231^DRHOC^ cells had significantly reduced invasiveness compared with MDA-MB-231^WT^ (p < 0.038 RHOC2 knockdown cells; p < 0.006 RHOC3 knockdown cells) and MDA-MB-231^pRevTRE^ control plasmid cells (p < 0.07 RHOC2 knockdown cells; p < 0.002 RHOC3 knockdown cells). An even greater reduction in invasiveness of the MDA-MB-231^DRHOC^ cells compared with the MDA-MB-231^WT^ cells was seen in response to hepatocyte growth factor (HGF/SF) (p < 0.009 RHOC1 knockdown; p = 0.004 RHOC2 knockdown; p = 0.00007 RHOC3 knockdown). The addition of doxycycline significantly improved the effectiveness of the ribozyme transgenes (p < 0.04 for all three Rho ribozymes), but did not improve the effectiveness of these knockdown cells when treated with HGF/SF (p > 0.1 for all three ribozymes).

**Conclusions:**

This data would indicate that targeting RhoC may be an effective way to reduce the invasive potential of human breast cancer cells.

## Introduction

Breast cancer is the second most common cause of cancer related deaths in women in the Western world. Patient prognosis is largely dependent on development of metastasis which is related to factors such as angiogenesis and the invasiveness and motile nature of breast cancer cells. Cell motility and migration are known to be regulated by the Rho family of GTPases through their effects on the actin cytoskeleton, cell-substrate adhesion as well as in membrane ruffling and lamellipodia extension [1-5].

Rho GTPases are members of the Ras superfamily of GTPases with at least 20 Rho proteins having been characterized. The Rho proteins cycle within the cell between the active GTP-bound form and the inactive GDP-bound form, the cycle being regulated by a number of activators and inhibitors. Three isoforms of Rho, Rho A, B and C, show a high degree of homology having over 85% amino acid sequence homology, however, they exhibit different cellular roles in the regulation of the cytoskeleton and cell motility [6]. RhoA functions in the regulation of actomyosin contractility, RhoB has been shown to regulate cytokine trafficking and cell survival, while RhoC appears to be important in cell locomotion.

The role of Rho in the development of human cancers has been investigated over the last 15 years or so. Over expression of RhoC was found to be correlated with prognosis in patients with pancreatic adenocarcinoma, with enhanced RhoC expression resulting in an increase in migration and invasion of pancreatic carcinoma cells [7, 8], while RhoA and RhoC up-regulation have been shown to be associated with tumor progression in ovarian carcinoma [9]. Over expression of RhoA has been observed in tumors from colon, breast and lung tissues, and was found to be significantly higher in grade III tumors than in grade I tumors in breast tissue, suggesting a link with tumor progression [10]. In prostate cancer it has been shown that RhoC is required for the invasive phenotype of PC3 cells [11]. Higher expression of RhoA, RhoC and ROCK have been correlated with increased invasion and metastasis in bladder cancer [12], with knockdown of RhoC inhibiting angiogenesis induced by hepatocellular carcinoma cells [13]. Microarray analysis has also shown increased RhoC levels in metastatic melanomas compared with primary tumors [14].

In breast cancer studies, RhoC has been identified as a highly specific marker in detecting tumors that developed metastases [15]. Over-expression of RhoC has been observed in the most aggressive form of breast cancer, inflammatory breast cancer, and is directly implicated in the control of the production of angiogenic factors in inflammatory breast cancer cells [16-19]. In vitro studies in rat mammary adenocarcinoma cells have shown that Rho family GTPases are differentially involved in motile functions and aspects of growth that correlate with tumorigenesis and metastasis [20].

We have previously reported a correlation between nodal involvement and metastasis with raised levels of RhoC, RhoG and Rho6 in breast tumor tissue together with significantly higher levels of RhoC and RhoG in patients who died of breast cancer [21]. This study aims to investigate the impact of targeting RhoC in human breast cancer cells by utilising ribozyme transgene technology and to assess its effect on cancer cell invasion.

## Materials and Methods

Human mammary cancer cells, MDA MB 231 were obtained from ECACC (the European Collection of Animal Cell Culture, Salisbury, England) and routinely maintained in DMEM F12 with 10% foetal calf serum. Recombinant human hepatocyte growth factor/scatter factor (HGF/SF) was a gift from Dr T Nakamura, Osaka University Medical School, Osaka, Japan. Matrigel (reconstituted basement membrane) was purchased from Collaborative Research Products (Bedford, Massachusetts, USA). A rabbit anti-human RhoC, and peroxidase conjugated anti-IgG were purchased from Santa-Cruz Biotechnologies (Santa Cruz, California, USA) and Sigma (Poole, Dorset, England, UK), respectively. A chemiluminescence detection kit for Western blotting and protein A/G conjugate were from Santa Cruz Biotechnologies. Transwell plates equipped with porous inserts (pore size 8 µm) were obtained from Becton Dickinson Labware (Oxford, UK). DNA restriction enzymes and T4 DNA ligase were obtained from New England Biological Laboratories (Hertfordshire, England). DNA gel extraction and plasmid extraction kits were sourced from Qiagen (Crawley, England).

### Construction of retroviral hammerhead ribozyme transgenes targeting human RhoC and generation of active viral hammerhead ribozymes

**Figure 1:**
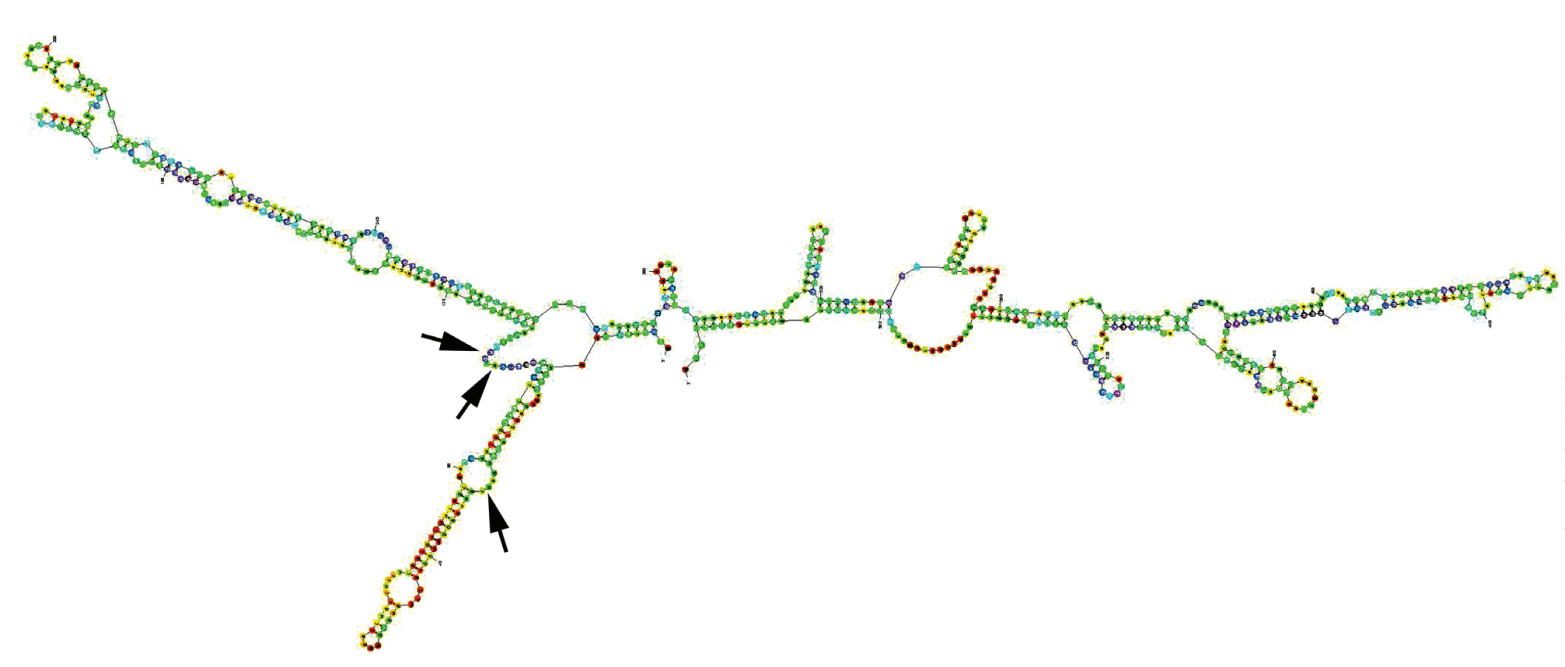
Secondary structure of human RHO-C mRNA, folded using Zuker’s programme [24]. Arrows: target sites for the ribozyme transgenes.

The procedure has been previously reported [22, 23]. Briefly, the secondary structure of human RhoC was generated using Zucker’s RNA mFold software [24] (Fig. 1). A panel of hammerhead ribozymes that specifically target *GUC* and *ATC* sites of human RhoC were generated using touch-down PCR using sets of RhoC ribozyme specific oligos. The products flanked by PstI and SpeI restriction sites were first cloned into a pZEO EcoSpe so that the transduction is downstream of the U1 promoter as we previous reported [22, 25, 26].

The correct inserts subsequently recloned into a retroviral vector pRevTre (REV Tet-On vectors, Clontech Laboratories, Palo Alto, California, USA), at a matched site. The successfully ligated plasmids were amplified in *E.coli* strain JM109 (Clontech). The direction and sequence were verified using a plasmid specific primer LXSNF-5’-CCCTTGAACCTCCTCGTTCGACC-’3 and a U1 specific primer 5’-GGATCCGCCAACCGAAAGT-3’ (UBAMHF) and 5’-GTACGATTAACAACTAAGA-’3 (UBAMHR).

### Retroviral packaging and transduction of cells

Plasmid, extracted and purified using a plasmid extraction kit (Qiafilter, Qiagen, Crawley, England), was introduced to a retroviral packaging cell line, PT67, using electroporation as previously described [27], followed by selection with G418 containing-medium for over 3 weeks. Viral titres from stably transfected PT67 cells were tested using NIH3T3 cells, and were found to be on average 8 X10^5^ cfu/ml. Active viral stocks were used to transduce MDA MB 231 mammary cancer cells in the presence of Polybrene (8 µg/ml final concentration). Each transduction lasted 24 hours and 3 consecutive tranductions were carried out. Transduced cells were subject to selection with G418 (Calbiochem, Nottingham, England), at 100 µg/ml for over 3 weeks in order to obtain stably transduced strains. These stably transduced and subsequently verified cells were designated the following names and are used through the text: MDA-MB-231^WT^- MDA MB 231 wild type; MDA-MB-231^pRevTRE^: MDA MB 231 transduced with pRevTRE empty vector; MDA-MB-231^DRHOC^ MDA MB 231 transduced with pRevTre-RhoC transgene.

### RNA preparation and RT-PCR

RNA from cells and tissues was extracted using an RNA extraction kit (AbGene Ltd, Surrey, England) and quantified using a spectrophotometer (Wolf Laboratories). cDNA was synthesised using a first strand synthesis with an oligo (dT) primer (AbGene, Surrey, UK). PCR primers for RhoC were as follows: RHOC-F1, 5’-AGCAGGGCAGGAAGACTATGA-3’, and RHOC-R1, 5’-TCAAGGTAGCCAAAGGCACTGAT-3’. The polymerase chain reaction (PCR) was performed using sets of primers with the following conditions: 5 min at 95°C, and then 20 seconds at 94°C, 25 seconds at 56°C, 50 seconds at 72°C for 36 cycles, and finally 72°C for 7 minutes. *ß-actin* was amplified simultaneously using the following primers: 5’-GCTGATTTGATGGAGTTGGA-3’ and 5’-TCAGCTACTTGTTCTTGAGTGAA-3’. PCR products were then separated on a 0.8% agarose gel, visualised under UV light, photographed using a Unisave camera (Wolf Laboratories) and documented with Photoshop software.

### In vitro invasion analysis

This was performed as previously reported and modified in our laboratory [28]. Briefly, transwell inserts (upper chamber) with 8 mm pore size were coated with 50 mg/insert of solubilized tissue basement membrane - Matrigel and air-dried, before being rehydrated. An amount of 20,000 cells of MDA-MB-231^WT^, MDA- MDA-MB-231^pRevTRE^ or MDA-MB-231^DRHOC^ were added to each well with, or without HGF/SF. After 72 hours, the non-invasive cells were removed with a cotton swab and the cells that had migrated through the membrane and adhered to the other side of the insert were fixed and, stained with 0.5% (w/v) crystal violet. Cells that had invaded and were stained with crystal violet were extracted with 10% (v/v) acetic acid and absorbance obtained at 540 nm using a multiplate reader.

Statistical analysis was carried out using Mann-Whitney U test and significant difference was taken at p < 0.05.

## Results

### Anti-RhoC ribozymes successfully knocked down RhoC expression from breast cancer cells

**Figure 2:**
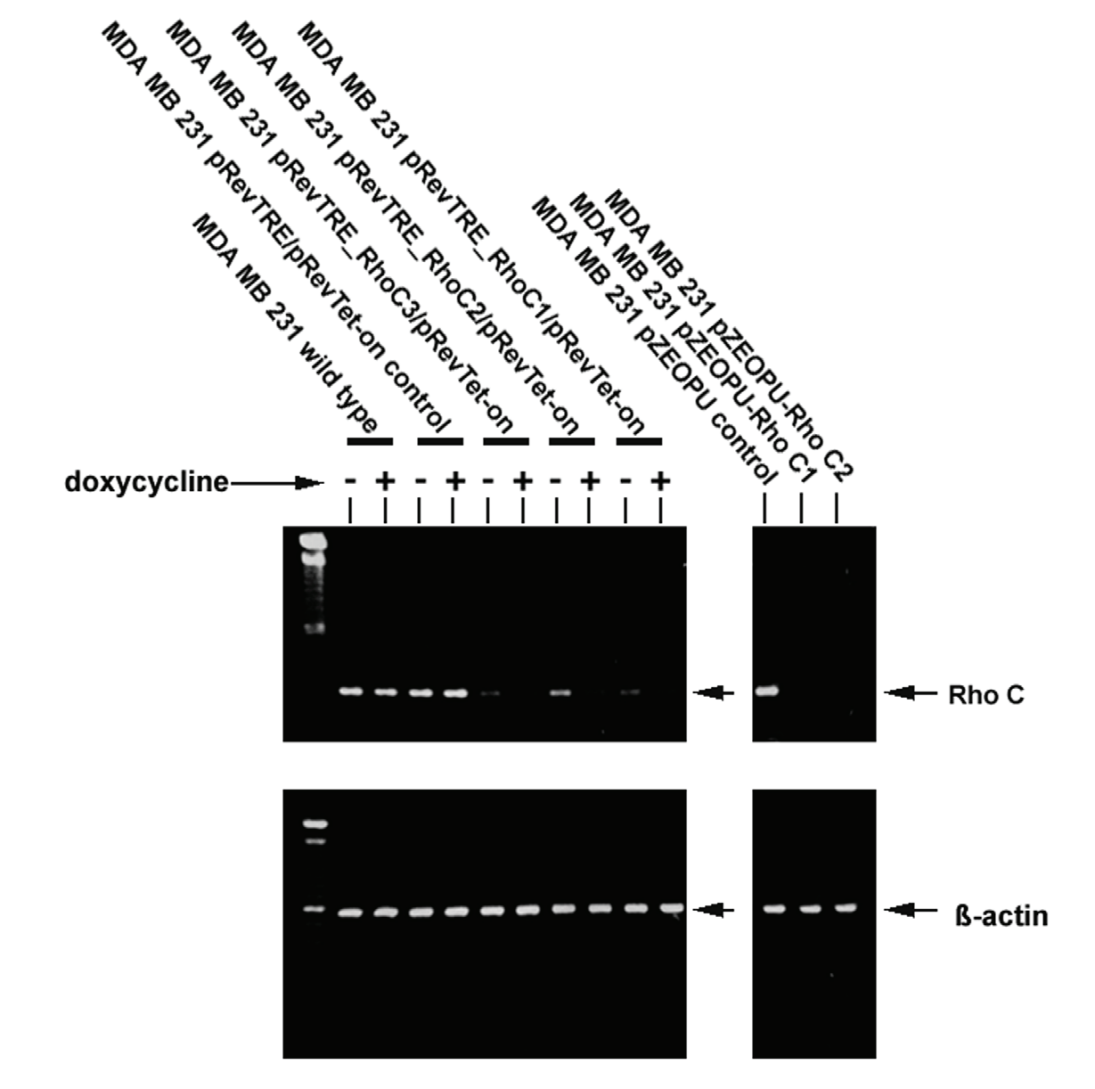
RT-PCR showing MDA-MB-231 wild type cells expressed RhoC transcript. Left: Retroviral transgenes pRevTre-RhoC successfully knocked down expression of the RhoC transcript from the MDA-MB-231 breast cancer cells. Right: anti-Rho-C transgenes constructed using the pEZO EcoSpe vector.

MDA-MB-231 breast cancer cells were shown to express RhoC as demonstrated by RT-PCR (Fig. 2). The ribozymes were first cloned in to the pZEO EcoSpe vector which had a U1 promoter. On transfection into the MDA MB 231 cells using the two transgenes that targeted the GUC sites, it was shown that both of the transgenes effectively knocked down the expression of RhoC from the cells (Fig. 2). The three transgenes were subsequently recloned into a regulated viral vector, pREV/TRE system which had the tetracycline response elements and responded to doxycline stimulation. The viral form of the three pRevTre-RhoC transgenes were used to transduce the breast cancer cells. The resultant sublines were designated MDA-MB-231^DRHOC1^, MDA-MB-231^DRHOC2^ and MDA-MB-231^DRHOC3^ and almost completely lost the expression of RhoC compared with the wild type, MDA-MB-231^WT^ and control plasmid cells, MDA-MB-231^pRevTRE^ (Fig. 2).

### Elimination of RhoC expression in breast cancer cells affects the in vitro invasiveness and migration of breast cancer cells

*In vitro* testing of the invasiveness of wild type, plasmid control and the RhoC knockdown cells showed that MDA-MB-231^DRHOC2^ and MDA-MB-231^DRHOC3^ cells had significantly reduced invasiveness compared with MDA-MB-231^WT^ (19.6 ± 3.12, p = 0.038 RHOC2 knockdown; 13.4 ± 1.68, p = 0.006 RHOC3 knockdown vs 26.2 ± 6.16 WT), similarly, both MDA-MB-231^DRHOC2^ and MDA-MB-231^DRHOC3^ showed reduced invasion compared with MDA-MB-231^pRevTRE^ control cells but this only reached a level of significance for the MDA-MB-231^DRHOC3^ cells (19.6 ± 3.12, p = 0.07 RHOC2 knockdown; 13.4 ± 1.68, p = 0.002 RHOC3 knockdown vs 25.25 ± 4.25 pRevTRE plasmid control). An even greater reduction in invasiveness of the MDA-MB-231^DRHOC^ cells compared with the MDA-MB-231^WT^ cells was seen in response to hepatocyte growth factor (HGF/SF) (35.25 ± 6.25, p = 0.009 RHOC1 knockdown; 31.5 ± 5.75, p = 0.004, RHOC2 knockdown; 11.8 ± 2.16, p = 0.00007, RHOC3 knockdown vs 56.75 ± 8.75 MDA-MB-231^WT^ cells.

**Figure 3:**
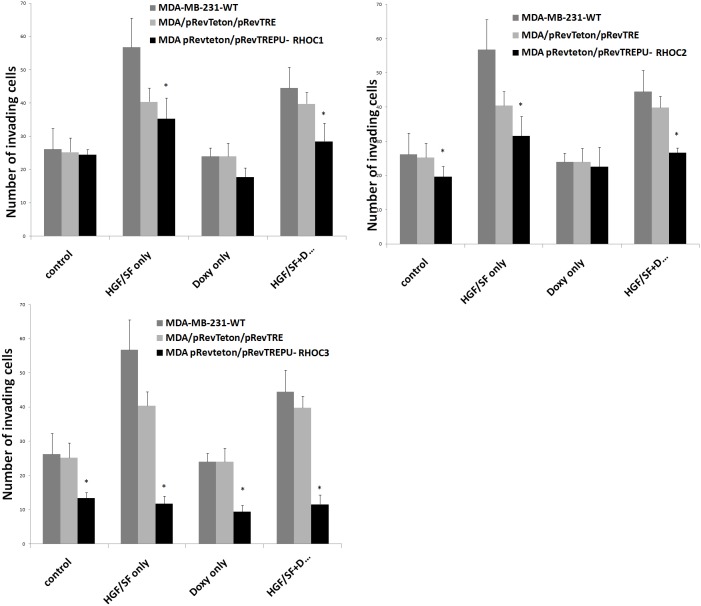
Graphical representation of the effect of a panel of ribozymes to RhoC on invasion of MDA-MB-231 breast cancer cells compared with MDA-MB-231^WT^ and MDA-MB-231^pRevTRE^ cells. Top left: MDA-MB-231^ΔRHOC1^; Middle: MDA-MB-231^ΔRHOC2^ and bottom: MDA-MB-231^ΔRHOC3^.

The pREV/TRE vectors carried the tetracycline response gene response element. Doxycycline was therefore used to fully switch on the expression of the transgene. The addition of doxycycline significantly improved the effectiveness of the ribozyme transgenes MDA-MB-231^DRHOC1^ (24.5 ± 1.5 RHOC1 knockdown vs 17.75 ± 2.75 RHOC1 knockdown + doxycycline, p = 0.01) MDA-MB-231^DRHOC2^ (19.6 ± 3.12 RHOC2 knockdown vs 22.6 ± 5.68 RHOC2 knockdown + doxycycline, p = 0.04) MDA-MB-231^DRHOC3^ (13.4 ± 1.68 RHOC3 knockdown vs 9.4 ± 1.92 RHOC3 knockdown + doxycycline, p = 0.02). However, the addition of doxycycline to the HGF/SF treated knockdown cells did not improve the ribozyme effectiveness (35.25 ± 6.25 RHOC1 knockdown + HGF vs 28.5 ± 5.5 RHOC1 knockdown + HGF + doxycycline, p = 0.13; 31.5 ± 5.75 RHOC2 knockdown + HGF vs 26.6 ± 1.52 RHOC2 knockdown + HGF + doxycycline, p=0.1; 11.8 ± 2.16 RHOC3 knockdown + HGF/SF vs 11.6 ± 2.72 RHOC3 knockdown + HGF/SF + doxycycline, p = 0.46) (Fig 3).

## Discussion

The development of metastasis in patients with breast cancer is of prime importance in patient prognosis. Detachment of tumor cells from the primary tumor and migration to a secondary site depends, in part, on changes in cell-cell adhesion, cell-matrix interactions and cytoskeletal changes leading to cell motility. The effects of the Rho proteins on the actin cytoskeleton point to a role as regulators in cellular motility and metastasis [2-4]. In this study, we have utilised ribozyme transgene technology to knock down the expression of RhoC in breast cancer cells in order to assess the impact of targeting RhoC on cancer cell invasion. RhoC is highly expressed in MDA-MB-231 cells, as seen in Figure 2. Our study has shown that the U1 promoter is sufficient in driving the expression of the anti-RhoC transgenes, resulting in an almost completely knocking down of the gene transcript in the breast cancer cells. Attempts to developing a viral version of the transgenes were also successful. It is interesting to note that the U1 promoter is also sufficient in this case, in that even without the induction by doxycycline, there has been substantial loss of the RhoC transcript. Doxycycline has further enhanced the effect in some case as shown in Figure 2.

The other purpose of the study is to establish a causal relationship between RhoC expression and in vitro invasiveness of breast cancer cells. Using the cells thus generated we have shown that loss of RhoC in the breast cancer cell MDA-MB-231^ΔRHOC^ is accompanied by the significant reduction in invasiveness of breast cancer cells *in vitro*. It is interesting to note that in the pRevTet-on ribozyme transgene system created, the knockdown was highly effective with or without doxycycline, indicating that the U1 promoter inserted in the pRevTre worked well, independent of tetracycline response elements. A further observation made from the study is that RhoC is an essential element in HGF regulated cellular invasion. Hepatocyte growth factor (otherwise known as scatter factor) is a protein factor that has a powerful effect on hepatocyte, cancer cells and endothelial cells [29, 30]. It is a powerful mitogen for normal hepatocytes, and an important regulator of angiogenesis [31-33]. On cancer cells, HGF mostly acts as an inducer of cell migration and invasion as demonstrated in previous studies [34-37]. It is evident from the present study that HGF markedly induced the invasiveness of breast cancer cells. Clearly, anti-RhoC1 and particularly anti-RhoC3 virtually abolished the effect of HGF-induced invasion. Together, it further indicates the pivotal role of Rho-C in HGF induced cancer invasiveness. HGF and its receptor are becoming recognised targets in cancer treatment [34, 35, 38, 39]. A few options are now available in this regard, including anti-HGF and anti-HGF antibodies, HGF specific antagonist and small molecule inhibitors [34, 35, 38-40]. A combined strategy in targeting both HGF and RhoC may be an attractive option in cancer therapies, an area that is under active investigation.

In conclusion, this study has shown that eliminating the expression of RhoC by ribozyme technology in MDA-MB-231 breast cancer cells reduces their *in vitro* invasiveness compared with wild type MDA-MB-231 cells. This, taken together with our previously reported work showing correlation between nodal involvement and metastasis with raised levels of RhoC, in breast tumor tissue and significantly higher levels of RhoC in patients who died of breast cancer [22], indicates that targeting RhoC may be an effective way to reduce the invasive potential of human breast cancer cells.

## Abbreviations

GTPases: Guanosine triphosphatases
HGF/SF: Hepatocyte growth factor/scatter factor
RT-PCR: Reverse transcription polymerase chain reaction
ROCK: Rho associated serine, threonine protein kinase
WT: Wild type
